# Vehicular influence on atmospheric concentrations and source apportionment of polycyclic aromatic hydrocarbons in some major cities in three regions of Ghana using epiphytic lichens

**DOI:** 10.1016/j.toxrep.2022.08.002

**Published:** 2022-08-23

**Authors:** Nathaniel Thompson, Joseph Kweku Adjei, John Kwesi Bentum, David Kofi Essumang, Godfred Odame Duodu, George Hadzi, George Alimo Adjei

**Affiliations:** a Department of Chemistry, University of Cape Coast, Ghana; b Ghana Atomic Energy Commission (GAEC), Legon, Accra, Ghana

**Keywords:** Lichens, Biomonitoring, Passive air sampler, PAHs, Source apportionment, PCA-APCS, APCS–ALM

## Abstract

The present study employed epiphytic lichens as biomonitor and passive air sampler for the assessment of fifteen (15) atmospheric polycyclic aromatic hydrocarbons (PAHs) in some major cities in three regions of Ghana. A total of 36 composite lichen samples were collected and analysed using Gas Chromatography - Tandem Mass Spectrometry (GC-MS-MS). The total PAH recorded ranged between 1909.9 ng/kg (A36) and 250,091.4 ng/kg (W15). Due to the inherent deficiencies in using a single source apportionment tool, multiple source apportionment methods including diagnostic ratios, principal component analysis/absolute principal component scores (PCA-APCS) and APCS with automatic linear model (APCS–ALM) were used to ascertain the source of PAHs in the lichens. The diagnostic ratios revealed a mix source of wood/grass and petrol/petroleum fuel combustion, with the major source ascribing to wood/grass combustion. The source apportionment confirmatory statistical test conducted with the PCA-APCS and APCS–ALM, were in good agreement with the diagnostic ratio. Both PCA-APCS and APCS–ALM suggested two significant sources (p < 0.0), with wood/grass combustion as the major (contributing 77.8%) and mix petroleum related sources being the other with 22.2% contribution of PAHs to the receptor sites. The study found PCA-APCS and especially APCS–ALM to be an effective statistical tool for PAH source apportionment in passive air samplers. To our knowledge, this is the first use of lichens for PAH monitoring in the country. Therefore, this study could serve as an inexpensive and real time bio-monitoring tool for air quality assessment in the African sub-region and the world at large.

## Highlights

• Epiphytic lichens was used as biomonitor for atmospheric PAH.
• Elevated B[*a*]P levels suggestive of eminent risk > 10^−4^ were recorded for most samples studied.
• The source apportionments conducted predicted petrol/petroleum fuel combustion as the major sources.
• This is widely potentially replicated study, using epiphytic lichens for the first time in Ghana to measure atmospheric PAHs.

## Graphical Abstract

**Figure:**
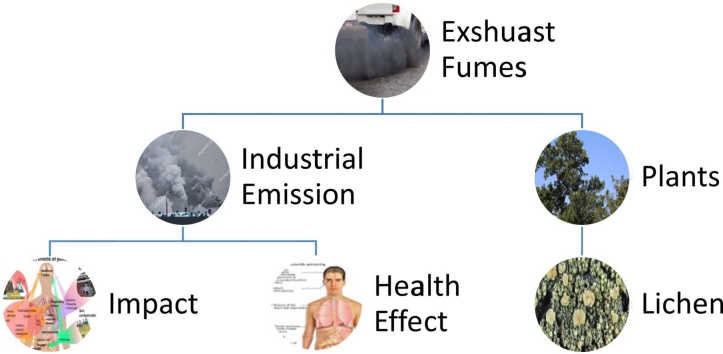

## 1 Introduction

Polycyclic aromatic hydrocarbons (PAHs), a class of pervasive organic pollutants, usually emitted into the atmosphere through the incomplete combustion and pyrolysis of organic matter such as wood and fossil fuel have become a global pollutant of concern [1], [2], [3]. PAHs are emitted into the environment by both natural and anthropogenic processes [1]. The sources of naturally produced PAHs include forest fires and volcanic eruptions, while human activities such as driving motor vehicles, industrial combustion process, waste incineration, thermal power generation, cooking and heating generate a bulk amount of PAHs [1], [4]. However, environmental conditions, geological factors, weather conditions, land use characteristics, cultural and customs of an area have a significant influence on the concentration of PAHs in the environment [4], [3].

Exposure to PAHs may result in several adverse human and environmental health effects [5]. For example, the International Agency for the Research on Cancer (IARC, 2016) has classified some PAHs as carcinogenic to humans (Group 1) or probably carcinogenic (Group 2 A). Other human health effects associated with exposure to PAHs include teratogenic and mutagenic effects as well as endocrine disrupting effects [1], [7], [6]. On the other hand, adverse effects including cell damage, reproduction, development, and immune defects have been observed in aquatic organisms and birds [8].

Due to their potential toxicity, the monitoring of PAHs in the environment plays a critical role in the environmental and human health risk assessment processes. With the exception of choosing a monitoring regime and method of analysis, the other primary challenge of environmental monitoring of PAHs is to distinguish between different sources in order to identify the main contributors of PAHs in an area of interest. In that regard, making use of passive samplers which are biomonitors and bioaccumulators is of great interest for the simplicity and rapidity with which it can be done [3], [9]. Consequently, the recent increase in the use of mosses, lichens, ferns, tree barks and other plants as passive collectors of atmospheric pollutants like PAHs [10], [11], [12], [13].

Specifically, lichens are symbioses of two organisms, a fungal mycobiont and a photoautotrophic photobiont, which present a very remarkable ability to uptake, accumulate and keep water, nutrients and contaminants directly from the air because they have no roots [14], [3]. In addition, lichens are ubiquitous and can be found on trees, rocks, soils and even on weevils and giant Galapagos turtles [15]. Moreover, they are tolerant and sensitive to a wide range of pollutants and weather conditions and hence can provide good spatial resolution data. Furthermore, their high surface-to-volume ratio, longevity and slow growth rate facilitate their accumulation of pollutants at varying levels [16], [17], [18], [3]. The mechanism of uptake of pollutants by lichens includes intracellular absorption, intracellular accumulation or deposition of particles [13]. Also, the transport of PAHs to biological receptors is reported to be affected by gas-particle partitioning (which depends on the volatility of each PAH), the surface area of particulate matter, seasonal factors (which are temperature dependent) and the source of the PAHs [19].

The outlook for air pollution in many developing nations including Ghana, is a cause for concern. The problem of air pollution is exacerbated by the increasing rate of e-waste burning as a result of the influx of electronic waste coupled with increasing vehicular traffic and indiscriminate bush burning [20], [21], [22], [23], [24], [25]. The smoke emanating from e-waste, plastics and other waste burning activities as well as fumes from vehicular and industrial sources, particularly from emerging industrialise cities, such as Kumasi and Takoradi in Ghana, is known to be detrimental to the health of the residents [28], [29], [21], [30], [31], [26], [27]. These smokes and fumes usually serve as a source of elevated levels of inorganic (e.g. Heavy metals) and organic pollutants such as PAHs [32].

This study, therefore, sought to employ epiphytic lichens for the monitoring of PAH levels in the atmosphere resulting from anthropogenic activities in three cities of Ghana namely; Kumasi, Takoradi and Cape Coast and also apportion their source. To our knowledge, this is the first use of lichens for PAH monitoring in the country. Therefore, this study could serve as an inexpensive and real-time bio-monitoring tool for air quality assessment in the African sub-region and the world at large.

## 2 Materials and methods

### 2.1 Study area

**Fig. 1:**
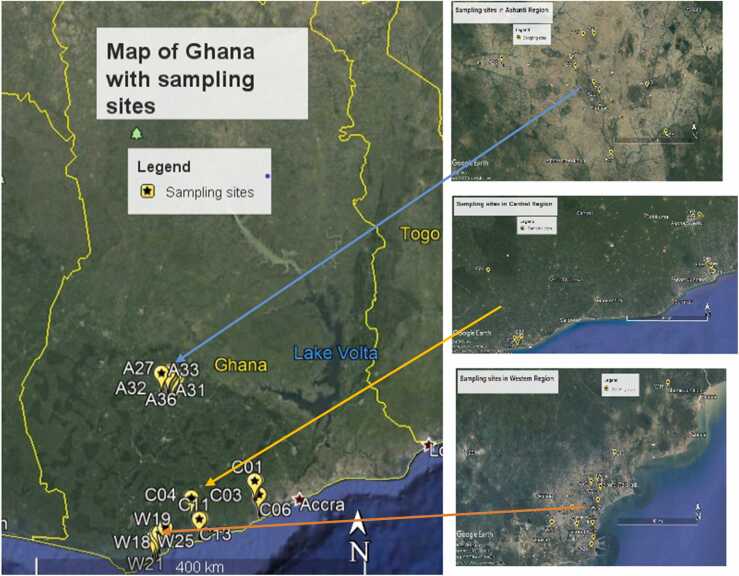
Map of Ghana showing sampling sites. Blue arrow points to sample sites in Ashanti region; orange arrow points to sites in Central region and brown points to sites in the Western region.

The Central, Western and Ashanti Regions are located in the coastal to the transitional forest zone of Ghana (Fig. 1). They experience a wet semi-equatorial climate with double maximum rainfall ranging between 214.3 mm in June and 165.2 mm in September. The average temperature ranges between a minimum of 20ºC and a maximum of 32ºC [33]. Prominent facilities like the Kwame Nkrumah University of Science and Technology (KNUST), the University of Cape Coast, the University of Education, Winneba, Kakum National Forest Reserve, the Suame Industrial Estate (Magazine) and the oil industry in Takoradi make them major commercial centres with increasing population growth [34].

### 2.2 Sampling

E*piphyti*c lichens were collected at random from selected areas of about 10 m^2^ in the Ashanti, Central and Western regions of Ghana. Sampling was done from August, 2014 to December, 2014. The lichens were carefully scraped from the tree barks using a stainless steel knife. All the samples collected in an area of 10 m^2^ were composite to form thirty six (36) major samples. To avoid contamination, the lichens were collected at about 1.5 m above the soil and neatly packed in air-tight amber glass and transported to the laboratory in a cooler. Samples which were considered relatively clean were also collected in the same manner at the Kakum National Park (a protected national forest reserve) in the Central Region of Ghana to serve as the control samples. Samples were kept frozen until processing.

### 2.3 Reagents and standards

Analytical grade reagents were used throughout the analysis. Standard mixture (QTM PAH Mix standard) containing sixteen PAHs, including naphthalene (NAP) acenaphthylene (ACY), acenaphthene (ACE), fluorene (FLU), phenanthrene (PHE), anthracene (ANT),fluoranthene (FLT), pyrene (PYR), benz(a)anthracene (B[*a*]A), chrysene (CHR), benzo(b + k)fluoranthene (B[b+k]F), benzo(a)pyrene (B[*a*]P), indeno(1.2.3.cd)pyrene (IND), dibenz(a.h)anthracene(DbA), benzo(g.h.i)perylene (B[*g*]P) and an alkylated PAH 2-bromonapthalene (BNAP) was purchased from Supelco Sigma-Aldrich Pty. Ltd (NSW, Australia).

All the reagents and reference standards used are the same as those reported earlier by Duodu et al. [1].

### 2.4 Sample preparation, extraction and clean-up

The stored samples were freeze-dried and then ground into powder using an agate mortar and homogenised.

About 2.0 g of each lichen sample was extracted using Soxhlet apparatus with 200 mL of acetonitrile for 24 h (EPA method, 3540). After extraction, extracts were concentrated by rotary vacuum evaporation and cleaned up using a C18 SPE column with 30 mL of acetonitrile as the eluting solvent. Subsequently, the extracts were concentrated using the rotary evaporator to about 5.0 mL and further concentrated with a gentle stream of pure N_2_ gas before reconstituted into 1.0 mL hexane. The cleaned extracts were then analysed for the 16-EPA PAHs using GC-MS/MS. Control samples were also treated and analysed in the same manner.

Prior to GC-MS/MS analyses, all the samples were spiked with an internal standards mix containing, naphthalene-D8, acenaphthene-D10, phenanthrene-D10, chrysene-D12 and perylene-D12 (EPA method, 3540).

### 2.5 GC-MS analysis of extracts

Analysis was done using Shimadzu GCMS-TQ8040 with splitless injection, MRM acquisition mode and EI ionisation in 22.83 min. The ResteK capillary column RXi-5MS (30 m × 0.25 mm × 0.25 m) was used with helium gas (purity > 99.999) as the carrier gas. The GC-MS/MS conditions were similar to that reported by Duodu et al. [1].

### 2.6 Quality control

At least five point calibration curves were used for quantification using the internal standard method. More than 10% of the samples were spiked with pure PAH standards to estimate the efficiency of the method used. Due to the lack of matrix blank for the recovery test, 2 g sea sand (solid blank) was spiked with 100 µL QTM PAH Mix standard (20 µg/mL) and treated as a sample. In the absence of matrix-matched certified reference material, the method validation and detection limit determination were similar to that reported by Duodu et al. [1].

### 2.7 Data analysis and statistics

Analysis of variance (ANOVA) at 95% confidence level, was employed for replicate analyses conducted. Initial source assessments of the PAHs were conducted using the diagnostic PAH ratios. Further, factor analysis, specifically principal component analysis (PCA) coupled with multiple linear regression and automatic regression analysis were also employed for source apportionment of the PAHs in the lichens using IBM SPSS Statistics 25 and SigmaPlot 14.0.

## 3 Results and discussion

### 3.1 Quality control results

All the data reported in this study were blank corrected. The recovery of PAH was evaluated using two different samples at different concentration levels as certified in NIST 1941b (high), and at 1.0 µg/g spiked sea sand (low). The mean recoveries of the PAHs investigated were > 80% at the high concentration level. Implying, good reproducible recoveries of PAHs in the certified material by the method with > 80% accuracy. Similarly, good reproducible mean recoveries (>80%) of most PAHs were also recorded at low levels. Here, the only exceptions were BgP and DbA with recoveries of 61% and 71%, respectively. In addition, the average recovery of Anthracene d-10 (surrogate standard) was 82%. The RF RSDs were generally < 15% for all PAHs.

The limit of detection (LOD) and limit of quantification (LOQ) for replicates (n = 7), were calculated to range from 0.01 to 0.21 ng/mL (LOD) and 0.04–0.70 ng/mL (LOQ) respectively [1], [35]. Results of quality control are similar to that published earlier by Duodu et al. [1].

### 3.2 Levels of PAHs in lichens

The mean concentrations of the fifteen (15) PAHs recorded in the lichen samples from the three regions (Central, Western and Ashanti) of Ghana are summarised in Table 1 and Table S2, S3 and S4, respectively in the supplementary data. Six PAHs (ANT, B[*a*]A, CHR, B[*a*]P, DbA and BgP) recorded values below the detection limits (BD) used in some samples from Western Region. However, all fifteen PAHs were detected in all samples from both Central and Ashanti Regions but for CHR in one sample from Central Region. The mean total concentrations of the 15 PAHs were between 213 and 251,225 ng/kg with an average of 15,483.6 ± 15,778 ng/kg (Table 1). In terms of the three regions, the mean concentrations of the PAHs ranged from 2664 ng/kg (C04) to 22,516 ng/kg (C11) (Table S2), 2687 ng/kg (W23) - 250,091 ng/kg (W15) (Table S3) and 1910 ng/kg (A36) to 14,964 ng/kg (A35) (Table S4), in Central, Western and Ashanti Regions, respectively.

**Table 1:** Mean concentrations of PAHs in lichen samples (Air) from some major cities in three regions in Ghana (ng/kg).

PAH	Min	Max	Median	Mean
NAP	123	3366	1873	1766.5 ± 760
ACY	6	1922	64	112.2 ± 103
ACE	4	906	37.5	67.5 ± 58
FLU	13	1357	73.5	113.1 ± 81
PHE	21	14,495	391.5	861.9 ± 857
ANT	BD	4198	102.5	215.4 ± 226
FLT	9	49,392	833	2218.8 ± 2626
PYR	18	52,524	1050.5	2479.1 ± 2780
B[*a*]A	BD	26,561	427	1199.5 ± 1409
CHR	BD	5704	85	237.5 ± 304
B[b+k]F	15	30,268	748.5	1503.6 ± 1598
B[*a*]P	BD	14,738	1547.5	2264.4 ± 1779
IND	4	20,591	330	1083.7 ± 1413
DbA	BD	7563	98.5	488.6 ± 734
BgP	BD	17,640	290.5	871.5 ± 1051
ƩPAHs	213	251,225	8646.5	15,483.6 ± 15,778

Samples labelled C04, W23 and A36 were taken from a forest reserve, a hospital and a residential area, respectively (see sampling point description in Table S1 of supplementary data) with little or no anthropogenic activities hence the low sum of PAHs concentration compared to C11, W15 and A35 which were taken from a commercial area close to a lorry or bus terminal, a commercial area at a busy road interchange and a commercial area, respectively known to generate high amounts of PAHs influenced by traffic emission [1], [4]. Similarly, the concentration of the sum of the six (6) carcinogenic PAHs (B[*a*]A, CHR, B[*b* + *k*]F, B[*a*]P, DbA, and INP) ranged between 19 and 105,425 ng/kg accounting for about 44% of the sum of the 15 PAHs. The concentration of PAHs recorded in this study was higher than that observed by Vitali et al. [3] ( 113,000– 183,000 ng/kg) but lower than that recorded by Landis et al. [11] ( 54,000– 2,778,000 ng/kg) and Shukla & Upreti, [36] ( 683,000– 33,720,000 ng/kg).

Generally, there was wide variation in the concentrations of the high molecular weight (HMW) thus four to six-membered ring PAHs compared to the low molecular weight (LMW) (two- and three-membered ring) PAHs (Table 1) and this may be attributed to multiple factors as reported earlier by Sett & Kundu, [13]. This is consistent with Sofowote et al. [19] assertion that transport of PAHs to biological receptors is affected by gas-particle partitioning, which is dependent on the volatility of each PAHs, and the surface area of particulate matter as well as the source of PAHs. Graney et al. [10] also observed that at longer distances HMW-PAHs were measured very close to analytical detection or quantitation limits. Generally, the uptake of PAHs by lichens is known to dependent on many factors [37], therefore the observed wide variation in HMW-PAHs concentrations in this study.

The concentration of the listed PAHs NAP, FLT, PYR, B[*a*]A, B[b+k]F, B[*a*]P and IND were considerably higher than the remaining PAHs. This could be attributed to the sources of the PAHs in the area. NAP and B[*a*]P as well as FLT, PYR, B[b+k]F, B[*a*]P and IND have been cited as strong markers for vegetation or biomass burning and diesel emissions, respectively [1].

The two-way ANOVA showed a statistically significant difference (p < 0.05) between the average total PAHs among the three regions. Similarly, statistically significant differences (p < 0.05) were found among the mean concentration of individual PAH molecules within a site. These results imply that the accumulation of PAHs by lichens differ significantly from various sites and thus, may depend on the sites and the anthropogenic activities around the sites [37]. This result is comparable to results obtained by Capozzi et al. [38] and Graney et al. [10].

### 3.3 Source assessment using the PAH diagnostic ratio

Diagnostic ratios of particular PAHs offer the opportunity to ascertain the origin of PAHs in the environment that may be due to pyrolytic (single source combustion of fuels), petrogenic (liquid fuels spills) and burning biomass or coal sources [39]. For instance, molecular weight (MW):178, ANT/(ANT + PHE) ratio is useful for identifying petrogenic sources, whereas MW:202, [FLT/(FLT + PYR)]; MW:228, [BaA/(BaA + CHR)] and MW:276, [InP/(InP + BgP)] are better for identifying pyrolytic sources [1], [40], [41], [42].

From Table 2, MW: 178, ANT/ (ANT + PHE) ratio ranged from 0 to 0.5. This could be inferred from Zhang et al. [43], Pies et al. [44] and Plachá et al. [45] in Table 2 that PAHs could be from both petrogenic and pyrogenic sources. Also, values recorded for MW:202, [FLT/(FLT + PYR)], MW:228, [BaA/(BaA + CHR)] and MW:276, [InP/(InP + BgP)] in this study were 0.02–0.7, 0 – 1 and 0.39 – 1, respectively. These values imply that the source of PAHs might be from mix sources of petroleum combustion, with vehicular emissions (both petrol emission and diesel emission), coal combustion and wood combustion [40], [46], [47], [42], [41]. However, coal is not a common fuel source in Ghana and may not be contributing factor. Other ratios [FLU/(FLU + PYR)] and [B[*a*]P/(BgP)] also ranged between 0.32 and 0.5 and 0–13.1, respectively. These also suggest petrogenic and fossil fuel combustion (both traffic and non-traffic emissions), with the non-traffic emission likely being wood and grass combustion [48], [49], [50], [40], [41], [51]. Consequently, it could be inferred from the diagnostic ratio that the PAHs in the study areas have predominantly combustion origin from liquid fossil fuel (related to vehicular emissions) and grass or wood combustion. The primary fuels for powering vehicles in Ghana are liquid fuels: petrol and diesel. The transport sector is responsible for 63% of the total petroleum consumption in Ghana, with the number of registered vehicles increasing by about 121% between the years 2000 and 2009 [52]. Similarly, burning of wood for charcoal, smoking of fish and cooking of food, solid waste burning and burning of vegetation as part of farming practices are known to contribute particulate matter to the atmosphere in Ghana [52], [23], [54], [53].

**Table 2:** Diagnostic ratios for PAHs source identification in lichen samples.

PAH ratio	Source type	Value range	This study	Reference
[ANT/(ANT + PHE)]	Petrogenic	< 0.1	0–0.5	[44], [45], [43]
	Pyrogenic/combustion	> 0.1		
[FLT/(FLT + PYR)]	Petrol emission	< 0.5	0.02–0.7	[47]
	Diesel emission	> 0.5		
[FLU/(FLU + PYR)]	Petrogenic	< 0.4	0.32–0.5	[48]
	Fossil fuel combustion	0.4–0.5		
	Grass, and wood	> 0.5		
	Combustion			
[B[*a*]A/(B[*a*]A + CHR)]	Coal combustion	0.2–0.35	0 – 1	[46]
	Vehicular emissions	> 0.35		
	Petrogenic/petroleum combustion	< 0.2		[42];
	Wood combustion	> 0.35		
	Wood combustion	1.2 – 5.0		[55], [50], [43],
				[44], [45]
[Ind/(Ind + BgP)]	Petrogenic/petroleum combustion	< 0.5	0.39 – 1	[40], [41], [42]
	Grass and wood combustion	> 0.5		
[B[*a*]P/(BgP)]	Petrogenic/traffic emission	> 0.6	0–13.1	[49], [50], [40], [51]
				[41]
	Non-traffic emission	< 0.6		
	Wood and grass combustion	1.2 – 5.0		

The results of the present study is comparable with that reported by Shukla et al. [56] in similar work, where combustion and mixed sources were the major and minor sources respectively.

### 3.4 Source apportionment of PAHs using principal component analysis coupled with absolute principal component scores (PCA-APCS) and automatic linear model (APCS–ALM)

The use of a multivariate technique such as principal component analysis/absolute principal component scores (PCA-APCS) and automatic linear model (APCS–ALM) to support diagnostic ratio in source apportionment helps to overcome the shortcomings of diagnostics ratio. It can quantitatively estimate the contribution of the various identified sources. PCA-APCS and APCS–ALM are very useful tools for source apportionment in environmental studies and details of the methods are described and applied in Adjei et al. [6], Pedersen et al. [57], Yang et al. [58], Jiang et al. [59] and Sofowote et al. [60]. The study further used these statistical protocols to ascertain sources identified by the use of the diagnostic ratio and also to identify the contributions of the sources it each receptor site.

**Fig. 2:**
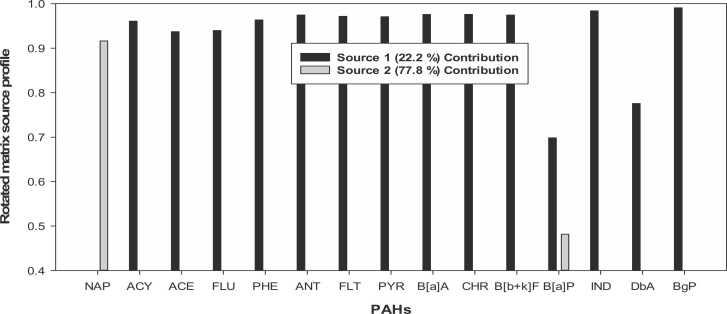
Source profile of PAHs in lichen samples from Central, Western and Ashanti Regions of Ghana.

**Fig. 3:**
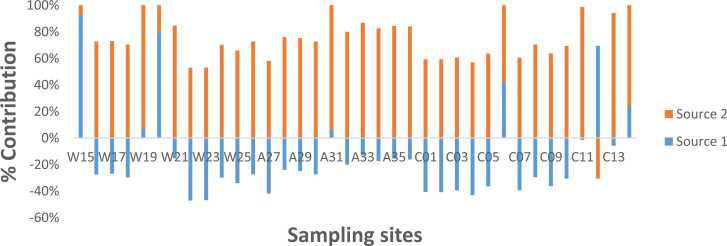
Source contribution plot showing the percentage contributions of the two sources.

The PCA-APCS was used to model the mean-centred PAH concentrations with variables below the detection limit replaced by one-half of its detection limits. The results of the PCA-APCS model are shown in Fig. 2, Fig. 3. The quality of the PCA-APCS model was evaluated using the correlation coefficient r^2^ of 0.999 and P < 0.001. The PCA extraction resulted in two (2) significant factor components after Varimax rotation (with Eigenvalues ≥ 1.0). The two factor components (Source 1 and Source 2) contributed about 94.1% of the total percent variance. Factor component one contributed 82.4%, whereas component two contributed 11.7% of the total variance recorded.

Source 1 contributed 22.2% of PAHs to lichen samples (Fig. 2) with significant loading of ACY, ACE, FLU, PHE, ANT, FLT, PYR, B[*a*]A, CHR, B[b+k]F, IND, DbA and BgP and minor contribution of B[*a*]P. The major presence of FLT, PYR, B[*a*]A, B[b+k]F, and BgP in this factor can be linked to fossil fuels combustion, particularly, diesel exhaust emissions [61], [1], [62]. Whereas PHE, ACY, FLU and ANT predominantly originate from petrogenic sources, particularly petroleum oil spillage, unburned fuel, lubricating oil and asphalt [63], [1]. While the 5- and 6-ring particulate phase PAHs IND, DbA and BgP are usually associated with petrol (gasoline) engine emissions [1], [64]. This source is therefore attributed to mixed petroleum related. This source was mainly seen at W15, W20, C06, C12 and C14 (Fig. 3). Site W15 is in a commercial area with vehicular traffic (Table S1), whereas W20 is residential area with a large vehicular volumes. Similarly, C06 is sited in a commercial area with vehicular traffic while C12 and C14 are in residential areas with large vehicular volume (Table S1).

The second source accounted for 77.8% of the total PAHs and was characterised by high NAP, and moderate B[*a*]P loadings. Both NAP and B[*a*]P have been used as a marker for biomass burning [1]. Therefore, this source was ascribed to biomass burning. This source was seen to impact almost all sites in the study areas (Fig. 3).

**Fig. 4:**
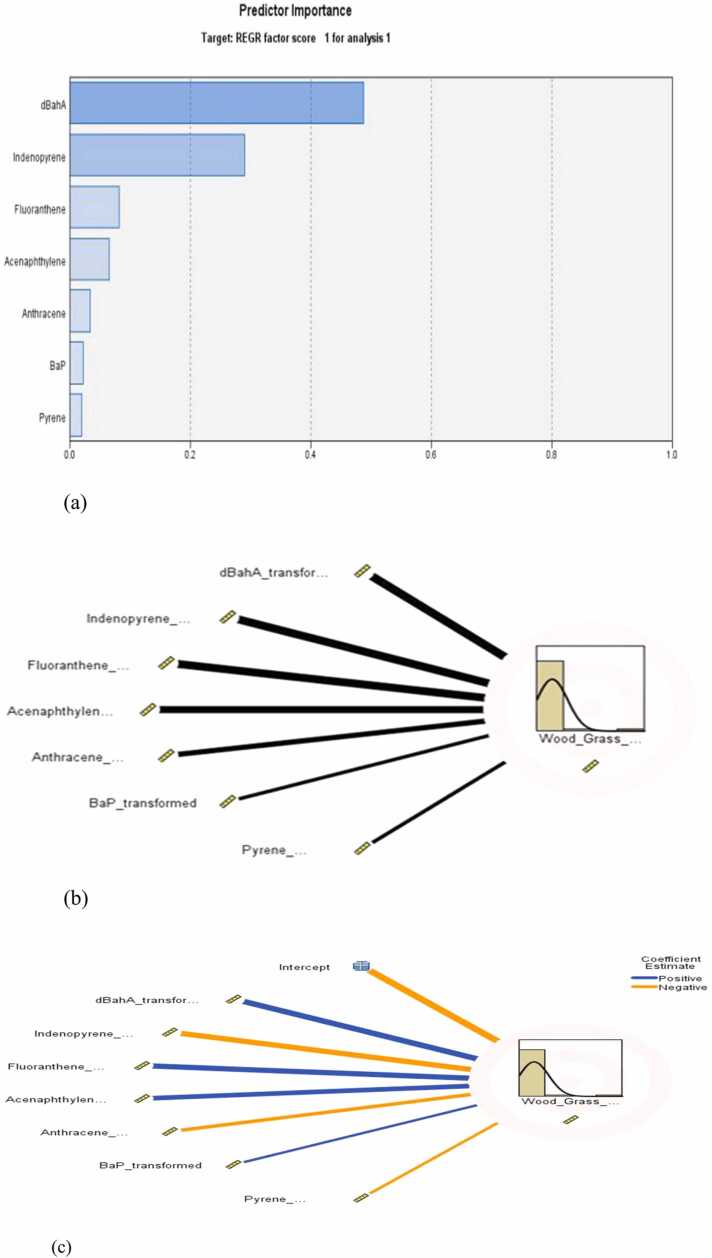
Automatic linear modelling graphs showing (a) the predictor importance (b) Effects and (c) Coefficients of significant predictors, for petroleum-related sources.

For exact and pictorial confirmation of the sources’ contributions to the levels of PAHs in the lichen samples analysed, the PCA-automatic linear modelling (APCS–ALM) was also performed. The results show that the model is about 94.5% accurate in estimating the contribution of the Factor component 1 (mixed petroleum-related sources) (Fig. 2) to the PAH levels in the lichen samples. Fig. 4(a) predicted that DbA is the most important predictor (predictor importance, pI = 0.49) and also the predictor with the highest statistical significant effect (p < 0.00). The second most important and effective predictor was IND (pI= 0.29, p < 0.0), but had a statistically significant negative influence (coefficient:−0.004, p < 0.0) on this source. The third most important and effective predictor of the petroleum-related sources was FLT (pI = 0.081; p < 0.0). The least important and the predictor with the least effect on this source was found to be PYR. The model report suggested that mixed petroleum-related sources may significantly and most importantly be predicted by the levels of DbA and to some extent by FLT and PYR. Duodu et al. [1] reported FLT, PYR, DbA as diesel markers.

**Fig. 5:**
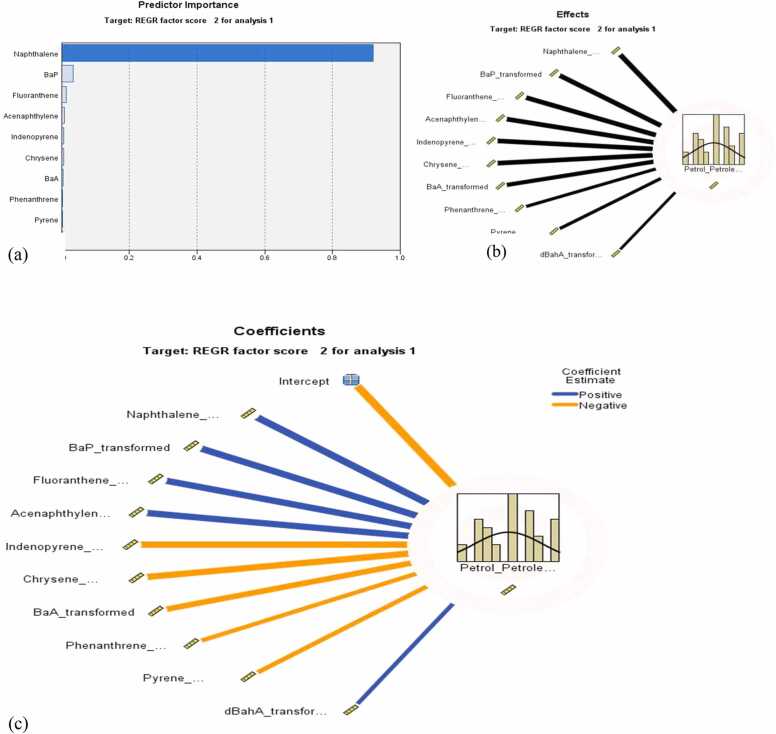
An automatic linear modelling graph showing (a) the predictor’s importance (b) Effects and (c) Coefficients of significant predictors for biomass burning sources.

The summary report from the APCS-ALM, again predicted that the model is 99.7% accurate in predicting the contribution of the second factor (biomass burning) to the PAHs levels recorded in the lichen samples. Fig. 5, predicted that NAP was the most statistically important predictor with the highest significant effect (pI = 0.921; p < 0.0). The second important predictor with the second highest effect in the model was B[*a*]P (pI = 0.034, p < 0.0), followed by FLT (pI = 0.017, p < 0.0). This model suggests that the levels of NAP in lichens may be used to significantly predict the biomass burning source of PAHs with an accuracy of 99.7%. Naphthalene (NAP) and B[*a*]P are known to be important loading components in biomass burning [63], [1], [65].

Though quite elaborated, the results obtained by Landis et al. [11] in similar study with lichens, where they found similar significant source using US EPA’s PMF 5.0, were comparable to the results obtained in the present study.

## 4 Conclusion

The study reveal that the lichens in the study area recorded higher concentrations of HMW-PAHs compared with LMW-PAHs. However, the lichen samples could be useful for accumulating LMW-PAHs with least deviations compared with HMW-PAHs with high deviations. The source apportionment results from the diagnostic ratio and both PCA-APCS, and APCS-ALM methods were in good agreement predicting two significant sources of mix petroleum-related sources and most importantly wood/grass combustion (biomass burning) source. The results suggest that lichen samples in the study area could be used as bio-monitoring/passive sampler of atmospheric PAH and also for source apportionment when coupled with statistical tools like PCA-APCS and APCS–ALM. Therefore, this study could serve as an inexpensive and real-time bio-monitoring tool for air quality assessment in the African sub-region and the world at large.

## Declaration of Competing Interest

The authors declare the following financial interests/personal relationships which may be considered as potential competing interests: David Kofi Essumang reports equipment, drugs, or supplies was provided by University of Cape Coast. David Kofi Essumang reports a relationship with University of Cape Coast that includes: employment. David Kofi Essumang has patent No Patent pending to No Licensee. Nothing more to declare.

## Data Availability

Data will be made available on request.
