# Supplementary material for: Vehicular influence on atmospheric concentrations and source apportionment of polycyclic aromatic hydrocarbons in some major cities in three regions of Ghana using epiphytic lichens
Source: Toxicol Rep. 2022 Aug 23;9:1691–9. doi: 10.1016/j.toxrep.2022.08.002 (PMC9742958; doi:10.1016/j.toxrep.2022.08.002)
Supplement: Supplementary file 1 — Supplementary material [file mmc1.pdf]

Supplementary data for:

Vehicular influence on Atmospheric Concentrations and Source Apportionment of Polycyclic Aromatic Hydrocarbons in some major cities in three Regions of Ghana using Epiphytic Lichens.

Nathaniel Thompson, Joseph Kweku Adjei[1,] John Kwesi Bentum[1], David Kofi Essumang[11]*, Godfred Odame Duodu[2], George Hadzi[1] and George Alimo Adjei [1]

[1]Department of Chemistry, University of Cape Coast, Ghana.

[2]Ghana Atomic Energy Commission (GAEC) Legon, Accra Ghana

Tables

Table S1: Sampling site description with coordinates

Code	Latitude	Longitude	Sampling Site	Description
C01	5°32'6.34"N	0°41'33.40"W	Swedru Municipal Hospital	Residential and commercial area
C02	5°21'44.44"N	0°37'51.71"W	University of Education, North Campus Lecturers Village	Residential area
C03	5°32'27.79"N	0°40'20.50"W	Swedru Secondary School	Residential area
C04	5°20'59.57"N	1°22'55.02"W	Kakum Forest Reserve	Forest reserve

Corresponding Author Essumang D. K. dessumang@ucc.edu.gh

C05	5°22'28.89"N	0°38'23.35"W	Efutu Municipal Assembly Traffic light	Commercial area
C06	5°23'0.63"N	0°38'33.60"W	Winneba Junction	Commercial area with vehicular traffic
C07	5°21'43.16"N	0°38'2.01"W	University of Education, North Campus	Commercial area
C08	5° 6'51.16"N	1°16'46.14"W	University of Cape Coast, East Gate	Garden with a road
C09	5°22'51.37"N	0°38'24.82"W	Ekem Ceramics, Winneba	Commercial area
C10	5° 6'5.28"N	1°17'3.39"W	University of Cape Coast, West Gate	Garden with a road
C11	5° 7'23.71"N	1°16'12.50"W	Pedu Junction	Commercial area with lorry or bus terminal
C12	5° 6'22.03"N	1°16'54.50"W	University of Cape Coast, Tech. Village	Residential area
C13	5° 6'59.23"N	1°17'37.23"W	University of Cape Coast, School of Science	Residential area
C14	5°20'43.94"N	0°37'31.91"W	University of Education, South Campus Old Administration Block	Residential area

W15	4°54'11.24"N	1°46'5.62"W	AGIP	Commercial area with vehicular traffic
W16	4°56'33.89"N	1°44'42.03"W	Regional Administration, Sekondi	Commercial area
W17	4°55'25.14"N	1°44'40.61"W	Afia Nkwanta Hospital	Mixed residential and commercial area
W18	5° 1'30.27"N	1°39'54.46"W	Shama Lorry Station	Commercial area with vehicular traffic
W19	4°57'53.77"N	1°43'33.25"W	Kojo Krom	Commercial area with rail lines
W20	4°56'5.74"N	1°44'30.17"W	Kweikrom	Residential area with large vehicular volume
W21	4°54'17.72"N	1°47'47.38"W	Apramdo	Residential area
W22	4°54'14.84"N	1°45'2.74"W	Monkey Hill	Elevated forest within a city
W23	4°53'7.15"N	1°45'7.07"W	European Hospital	Residential and commercial area
W24	4°55'2.77"N	1°46'24.79"W	TADISCO	A School

W25	4°56'22.39"N	1°45'7.40"W	Fijai	A School
W26	4°53'29.46"N	1°44'59.07"W	Harbour Road	Commercial area near a harbour
A27	6°43'16.28"N	1°37'59.45"W	Suame Magazine	Commercial area with vehicular traffic
A28	6°43'18.20"N	1°37'19.44"W	Tafo Cemetery	Road with vehicular traffic
A29	6°40'23.34"N	1°36'59.58"W	Tafo Nyhiaeso	Residential area
A30	6°37'10.95"N	1°36'18.38"W	Sokoban Wood Village	Commercial area
A31	6°40'41.07"N	1°34'6.46"W	KNUST Okodie Road	Residential area
A32	6°42'1.27"N	1°42'57.11"W	Abuakwa	Commercial area with vehicular traffic
A33	6°42'6.39"N	1°38'43.55"W	Sofoline	Commercial area with vehicular traffic
A34	6°40'47.95"N	1°37'19.53"W	Ridge Police Station	Residential and commercial area

A35	6°38'16.10"N	1°32'57.50"W	Aprabon	Commercial area
A36	6°41'38.37"N	1°38'27.16"W	South Suntreso	Residential area

Table S2: Mean Levels of PAHs in lichens samples from Central region of Ghana (ng/kg)

Compound	C01	C02	C03	C04	C05	C06	C07	C08	C09	C10	C11	C12	C13	C14
NAP	1657	1130	1112	1240	1342	589	3366	2903	743	3164	2167	135	1134	469
ACY	30	17	22	20	34	82	60	92	20	98	104	6	75	43
ACE	19	37	14	23	21	38	53	58	13	70	79	11	28	27
FLU	49	32	31	50	44	71	115	109	25	123	159	16	51	40
PHE	161	134	143	21	192	476	209	568	117	400	553	128	351	257
ANT	49	27	37	19	49	179	39	93	36	108	172	129	116	89
FLT	205	181	332	167	384	1283	327	803	384	872	1910	133	1165	784
PYR	230	217	372	204	468	1509	418	1175	455	1099	2199	130	1332	889
B[a]A	188	125	221	108	231	724	175	411	226	533	1118	717	644	414
CHR	21	17	28	11	35	120	49	83	43	109	BD	144	115	97
B[b+k]F	344	266	466	222	536	851	407	719	490	890	1406	926	855	558
B[a]P	471	523	713	260	1518	2887	2151	5340	874	3626	10666	432	2272	821
IND	227	73	175	128	150	493	228	478	124	605	916	7012	440	266
DbA	189	85	44	99	34	150	95	145	23	177	256	7563	130	72
BgP	190	114	215	93	188	506	243	421	116	547	812	3019	465	243
Mean total	4031	2977	3924	2664	5225	9956	7942	13411	3687	12443	22516	20520	9173	5066

Table S3: Mean Levels of PAHs in lichens samples from Western region of Ghana (ng/kg)

Compound	W15	W16	W17	W18	W19	W20	W21	W22	W23	W24	W25	W26
NAP	3177	2322	2472	3259	2329	151	852	2861	1932	1952	1821	2179
ACY	1922	68	73	95	56	14	33	9	14	102	59	108
ACE	906	46	42	49	216	21	20	19	13	66	31	78
FLU	1357	82	92	98	223	39	59	51	34	105	65	131
PHE	14495	1082	635	636	1664	1325	601	100	229	281	371	376
ANT	4198	133	153	157	278	68	66	BD	14	97	83	135
FLT	49392	1377	1483	1793	2308	1214	1002	9	86	456	664	781
PYR	52524	2038	1763	2043	2077	2124	1101	18	145	494	713	880
B[a]A	26561	533	852	954	963	262	388	BD	BD	366	391	440
CHR	5704	108	132	143	156	50	72	13	BD	55	66	87
B[b+k]F	30268	635	972	1119	943	263	443	15	51	1050	1015	612
B[a]P	14738	1577	1709	1811	1399	204	770	BD	77	558	701	1673
IND	20591	378	544	709	544	105	239	4	48	318	223	301
DbA	6618	104	145	188	156	21	58	BD	14	97	68	70
BgP	17640	368	539	729	475	98	205	BD	34	264	238	269
Mean total	250091	10877	11609	13786	13790	5973	5913	3098	2687	6299	6507	8154

Table S4: Mean Levels of PAHs in lichens samples from Ashanti region of Ghana (ng/kg)

Compound	A27	A28	A29	A30	A31	A32	A33	A34	A35	A36
NAP	2052	1821	1808	2331	1471	2224	1925	666	2716	123
ACY	35	83	80	76	100	97	100	44	158	11
ACE	16	36	43	49	57	59	56	19	94	4
FLU	52	76	89	93	113	117	94	33	142	13
PHE	524	516	383	574	1165	575	633	315	691	148
ANT	31	129	117	135	193	139	172	64	232	20
FLT	227	1067	863	1372	1458	1170	1563	658	1707	298
PYR	385	1288	1002	1558	1890	1354	1874	809	2118	354
B[a]A	28	621	512	733	657	613	937	343	1030	163
CHR	6	102	106	132	134	171	163	60	195	23
B[b+k]F	453	781	778	785	1001	1039	1133	463	1197	178
B[a]P	457	2684	2644	2680	3380	3800	3523	1030	3225	326
IND	200	348	351	342	437	577	456	197	678	107
DbA	57	108	101	75	98	160	101	37	201	51
BgP	171	312	319	354	413	427	497	182	576	92
Mean total	4695	9972	9194	11291	12573	12523	13225	4918	14964	1910
